# A sclerite-bearing stem group entoproct from the early Cambrian and its implications

**DOI:** 10.1038/srep01066

**Published:** 2013-01-17

**Authors:** Zhifei Zhang, Lars E. Holmer, Christian B. Skovsted, Glenn A. Brock, Graham E. Budd, Dongjing Fu, Xingliang Zhang, Degan Shu, Jian Han, Jianni Liu, Haizhou Wang, Aodhán Butler, Guoxiang Li

**Affiliations:** 1 Early Life Institute, State Key Laboratory of Continental Dynamics, Northwest University, Xi'an, 710069, China; 2 Uppsala University, Department of Earth Sciences, Palaeobiology, Villavägen 16, SE-752 36, Uppsala, Sweden; 3 Department of Palaeozoology, Swedish Museum of Natural History, Box 50007, SE-104 05, Stockholm, Sweden; 4 Department of Biological Sciences, Macquarie University, New South Wales 2109, Australia; 5 LPS, Nanjing Institute of Geology and Palaeontology, Chinese Academy of Sciences, Nanjing, 210008, China

## Abstract

The Lophotrochozoa includes disparate tentacle-bearing sessile protostome animals, which apparently appeared in the Cambrian explosion, but lack an uncontested fossil record. Here we describe abundant well preserved material of *Cotyledion tylodes* Luo et Hu, 1999, from the Cambrian (Series 2) Chengjiang deposits, reinterpreted here as a stem-group entoproct. The entoproct affinity is supported by the sessile body plan and interior soft anatomy. The body consists of an upper calyx and a lower elongate stalk with a distal holdfast. The soft anatomy includes a U-shaped gut with a mouth and aboral anus ringed by retractable marginal tentacles. *Cotyledion* differs from extant entoprocts in being larger, and having the calyx and the stalk covered by numerous loosely-spaced external sclerites. The description of entoprocts from the Chengjiang biota traces the ancestry of yet another lophotrochozoan phylum back to the Cambrian radiation, and has important implications for the earliest evolution of lophotrochozoans.

The extant members of the Phylum Entoprocta are all small sessile suspension-feeders that consist of a calyx, stalk and basal attachment, but lack a skeleton^1^. The only uncontested fossil entoproct comes from the late Jurassic^2^. The Cambrian *Dinomischus* from the Burgess Shale and Chengjiang biotas was originally suggested to belong within the Entoprocta^3^,^4^; however, the supposed filter-feeding apparatus of *Dinomischus* is composed of stiff bracts and lacks evidence of the flexible tentacles that are characteristic of extant entoprocts^2^,^5^. Here we report for the first time a probable stem entoproct, originally described as *Cotyledion tylodes* Luo et Hu, 1999 in Problematica^6^, from the Chengjiang Konservat-Lagerstätte [about 520 Ma], Kunming, Yunnan, China. The entoproct affinity is supported by the presence of a lower fleshy stalk and an upper cup-shaped calyx containing a U-shaped gut with mouth and projected anal opening, both of which are surrounded by a marginal crown of flexible and foldable tentacles. This combination of characters is closely comparable with that of extant Entoprocta. However, *Cotyledion* differs from all crown entoprocts in having a relatively large size (8–56 mm, compared to a typical extant entoproct of 0.1–7.0 mm). The most notable difference, however, is that the entire outer surface of the body of *Cotyledion* is covered by numerous closely-spaced, pyrite-coated sclerites that originally might have been mineralized. In conjunction with studies of articulated tommotiids more recently interpreted as members of the brachiopod/phoronid stem group^7^,^8^,^9^, this sclerite-bearing tentaculate animal demonstrates that external sclerites may be more widely distributed within stem lophotrochozoans than previously suspected^10^, raising the possibility that sessile extant lophotrochozoan phyla probably were rooted in the earliest Cambrian Small Skeletal Fauna (SSF).

## Results

### Systematic Palaeontology

Total group Lophotrochozoa

Stem group Entoprocta

*Cotyledion tylodes* Luo et Hu, 1999

### Referred Material

ELI-C 0001-0418, all the specimens are housed in the Early Life Institute and Key Laboratory of Continental Dynamics, Northwest University, Xi′an, China (Prefix: ELI).

### Locality and Stratigraphy

Heilinpu (Chiungchussu or Qiongzhusi) Formation, Yu'anshan Member (*Eoredlichia-Wutingaspis* Trilobite Biozone, ordinarily correlated with the late Atdabanian or Botoman Stage in Siberia)^6^, Cambrian Stage 3. A total of 162 specimens come from the Jianshan Section at Haikou town, about 48 km south of Kunming, and the remaining 256 are mostly from the Erjie section, about 24 km southwest of Haikou.

### Revised diagnosis

Sessile bipartite body, comprising an upper cup-shaped calyx and lower stalk terminated by an attachment disc, completely covered by mineralized or possibly sclerotized, oval sclerites; internal anatomy with U-shaped alimentary canal: comprising a narrow anterior esophagus, extending from a recessed mouth cavity and followed by an enlarged stomach and a recurved narrow intestine, terminating in an anal opening. Both mouth and anal opening are enclosed within the same tentacle ring. Tentacles, slender, protruding from the upper margin of calyx. Stalk variable in length and terminated by knob-shaped holdfast for attachment to hard substrates.

### Description of new material

Continued collecting has produced important new data on *Cotyledion tylodes*^11^ since the original report and brief description of *Cotyledion*^6^,^12^. All available material from two localities can unequivocally be referred to the same species. The majority of *C. tylodes* specimens occur as compressed moulds that replicated the internal or external surface of this animal; some individuals were strongly flattened and preserved as reddish-brown flattened films with exceptionally preserved details of internal anatomy (n = 26).

**Figure 1:**
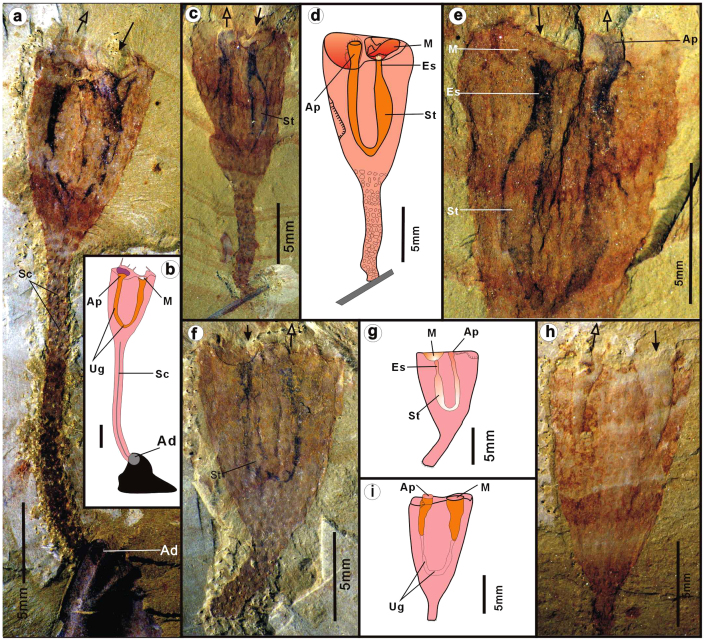
*Cotyledion tylodes* Luo & Hu 1999 from the Cambrian Stage 3 Chengjiang fauna (Yunnan, China). The hemispheric buccal cavity with basal mouth (anterior) and elongate anal papillae (posterior) marked with solid arrows and hollow arrows, respectively. (a), ELI-C-0369, a complete flattened specimen attached by long stalk to exoskeleton of trilobite; note the circular attachment disc (Ad); (b), interpretative drawing; (c), ELI-C-0359A, a laterally compressed specimen attached to gena of a trilobite, showing U-shaped gut with 3-D buccal cavity and enlongate anal tube enclosed inside apertural cavity; (d), interpretative drawing; (e), ELI-C-0359A, counterpart of C, close-up view of the calyx showing the U-shaped gut with buccal cavity and anal papillae; (f), ELI-C-0334A, a flattened specimen showing U-shaped digestive tract; (g), compare to f; (h), ELI-C-0338A, showing mud-filled buccal cavity and anal tube; (i), Compare to h; Scale bars are indicated. Abbreviations: Ad, attachment disc; Ap, anal papilla; Bc, buccal cavity; Cc, the interior cavity of calyx; Es, esophagus; In, intestine; M, mouth; Sc, the central cavity of stalk; St, the distended stomach; Ug, the U-shaped gut.

**Figure 2:**
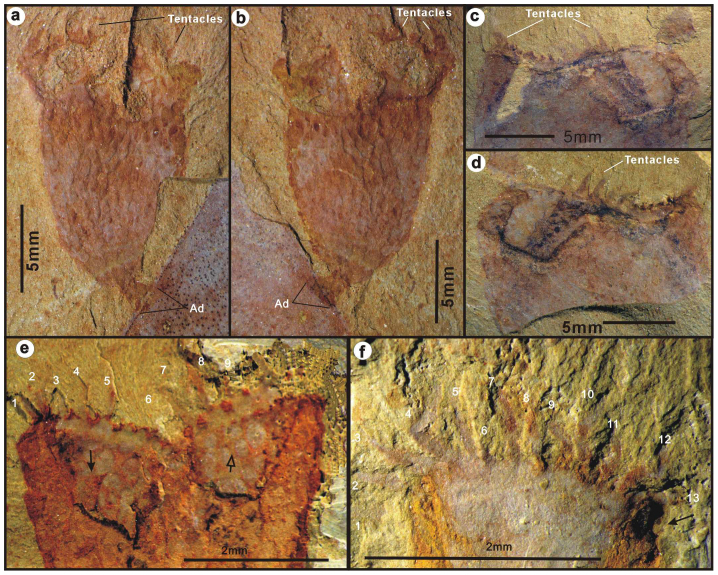
*Cotyledion tylodes* Luo & Hu 1999 from the Cambrian Stage 3 Chengjiang fauna (Yunnan, China). (a–b), ELI-C-0340AB, part and counterpart, a small individual attached by a distal attachment disc (Ad) onto a big exoskeleton of unknown affinity; note the oval tentacle crown. (c–d), ELI-C-0358AB, part and couterpart, top view of a compressed and distorted tentacle crown ringed by tentacle stubs. (e), ELI-C-0107A, Internal mould of the banded tentacle crown with dotted impression of tentacles marked with numbers; note the putative buccal cavity (solid arrow) and anal vestibule (hollow arrow); (f), ELI-C-0361A, External mould of a crown of tentacles marked with numbers.

The body of *C. tylodes* is shaped like a goblet, composed of an upper cup-like calyx and a lower cylindrical stalk (Fig. 1a–h). The calyx is usually compressed in the fossil state, with an elliptical transverse section (Fig. 2a–d, Fig. S2a, S4a). Calyx length ranges between 4.3 mm and 42 mm (mean 13.3 mm; n = 292), and the maximum width ranges from 3 mm– 23 mm with mean of 8.82 mm (n = 292) (Fig. S1). The ratio of maximum height/width of the calyx ranges from 1.05 to 2.25 (mean 1.61) (Table S1). The interior of the calyx, in a few cases, contains substantial sediment infill, apparently more pronounced in the upper part (Fig. S2a). This sediment infill suggests the interior originally contained a body cavity, probably accommodating the visceral organs, such as the digestive tract.

As seen in specimens ELI-C 0359B (Fig. 1c, d), a recurved tube, delineated by two undulating parallel curved black lineations, is interpreted as the U-shaped digestive tract. The U-shaped gut occurs either as a flattened tubular impression with blackish pigment (Fig. 1a–g), or as a partly (Fig. 1h–i) or completely mud-filled tube with some relief (Fig. S2b–d). A distended kidney-shaped region is interpreted to represent the stomach, which makes up the lower 2/3 of the U-shaped gut (Fig. 1c–e). Anterior to the stomach is a dark-stained tube, interpreted as an esophagus, which leads anteriorly into a hemi-spherical chamber filled with sediment (Fig. 1c–d; Fig. S2b,f,g). This is also readily observable in the counterpart specimen ELI-C 0359A (Fig. 1e), where the chamber is interpreted as a funnel-like buccal cavity (Fig. 1c–e). Beyond the bulbous stomach, the digestive canal shrinks to become a short tapering tube; it then recurves, forming a tubular intestine that extends upward reaching the main aperture of the calyx. The digestive canal terminates at a slightly projected anal opening, evidently located in the apertural vestibule (Fig. 1d). The buccal cavity and anal opening are often obscured by in-filled sediment within the apertural vestibule (Fig. 1a–b). In several flattened specimens (Fig. 1f, h), the funnel-like buccal cavity and anal opening form separate units, each of which possesses relief and are in-filled by sediment. The cavity is also encircled by the upper margin of the calyx. The mouth and anus are at opposite sides of the long axis of the suboval vestibule, here regarded as marking the anterior and posterior respectively (Fig. 1a–i).

A circle of tentacles originate from the margin of the calyx, preserved as reddish, flat banded impressions within the fine-grained bedding planes (Fig. 2; Fig. S2e–g). The bases of the tentacles are connected to the calyx margin by an undulating constriction of reddish, indented, membranous band (Fig. 2c–d). Each tentacle originates from an indentation in the membrane and protrudes outwards up to 1.6–3.2 mm in length (Fig. 2e, Fig. S3). We interpret each of these reddish indentations to represent the insertion point of an original tentacle, although only 9 well-preserved tentacles can be recognized with any certainty in Fig. 2e. In one, possibly anterior-posteriorly compressed specimen, with some relief, 13 separate tentacles stretch outward to form a semicircle (Fig. 2f). The tentacles are evenly spaced, with approximately six tentacles per millimeter. The maximum estimated number of tentacles based on available marginal space is 34^12^ (Fig. S3). The tentacles appear to be parallel-sided, and project straight up from the calyx in most cases (Fig. 2). In a few specimens (Fig. S4a), the tentacles evidently radiate outward away from the calyx aperture. In contrast, in some specimens curved tentacles appear to be retracted, and are converged toward the center of the calyx (Fig. S3c, S4b–c, I; also see Clausen et al. 2010: Fig. 2.3–4,^11^). In these specimens, the tentacle membrane bands were apparently tightened resulting in the maximum diameter of the calyx occurring slightly below the top of the apertural margin (Fig. S4b–c).

The high variability of calyx height to width ratio is a direct result of differing compaction of this cup-like structure. The ovoid and conical shape is demonstrated clearly by specimens compacted along the direction of the stalk, showing an ovoid tentacle crown (Fig. S4a, c). As seen in specimen ELI-C 0370, the calyx was compressed along the mouth-anus direction, and thus exhibits a parallel-sided tongue-shaped contour (Fig. S4d).

The central base of the calyx connects directly to an extended stalk, the distal end of which is normally attached to exoskeletal debris of other organisms (Fig. 1a, c; Fig. S4d–h). The animals were singly or multiply attached to a common hard substrate provided by other bioclasts (Figs. 1a–d, 2a,b; Fig. S4 d–h). In several specimens, the terminal end is free of bioclasts, revealing a knob-shaped attachment disc (Fig. S4j–k). The length of the stalk varies from 2.7– 38.1 mm, with a diameter between 0.8–3 mm (see: Supplementary Table. S1). The central section of some stalks contains a darkish sinuous lineation (Fig. 1a–b; Fig. S4g, l), here interpreted as a lumen extending from the main calycal cavity.

**Figure 3:**
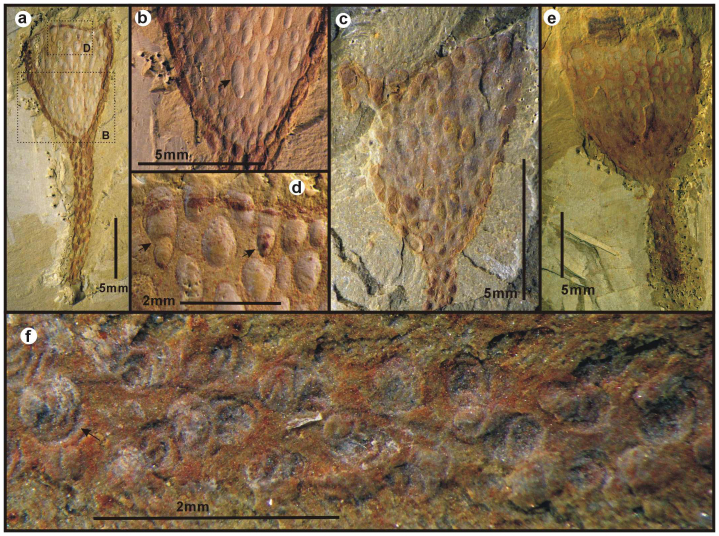
Sclerites on the calyx and stem of the stem entoproct *Cotyledion tylodes* Luo & Hu 1999 from the Cambrian Chengjiang fauna (Stage 3) of Yunnan, China. (a), ELI-C-0211A, dashed boxes indicate positions of b and d, note the seemingly increased arrays of sclerites; (b), details of a indicated; note the elongate sclerites marked by an arrow; (c), ELI-C-0344B, an external mould; showing the sclerites in some distinct relief; (d), Enlargement of a indicated, showing the merged two sclerites indicated by two arrows; (e), ELI-C-0186A, an interior view of calyx showing the impressions of sclerites; (f), ELI-C-0069A, close-up view of sclerites on the stalk to show the concentric lamellae.

The outer surface of the calyx and stalk is completely covered by loosely-spaced, rounded to elliptical external sclerites that have a quite pronounced relief (Fig. 3a–e). The sclerites are almost invariably composed entirely of pyrite pseudomorphs (Table S2), most commonly framboids (Fig. S5c–f). The length of sclerites is estimated to range from 0.2 mm to 2.8 mm, with a mean of 0.7 mm (n = 769), although most sclerites are strongly deformed and sometimes compressed (Fig. 1a,c,f,h). In general, they are seemingly arranged in longitudinal rows. The number of sclerites in the calyx varies from 39 to 203 in the available specimens. The sclerites in calyx and stalk are comparable in size and width. Arrays of sclerites radiated from the base of calyx, are seemingly in alignment with those on the stalk, where 3 rows of sclerites are usually recognizable (Fig. 3a–c). Sclerite rows evidently increased in number as the conical calyx grew. At least 3 pairs of sclerite rows radiate from the base of the calyx, and 2 mm away from the base new rows of sclerites arise and intervene between the earlier ones (Fig. 3a,b). The largest sclerite (Fig. 3b), probably representing two sclerites merging in the same row, is found in the uppermost part of the calyx (Fig. 3d). In the internal moulds of calices, the sclerites are expressed as loosely spaced shallow imprints (Fig. 3e, Fig. S2e–f, S5a–b). As seen in Fig. 3f, the outer surface of individual sclerites is marked by a series of tightly spaced lineations that we consider to represent growth lines and evidence for accretionary growth. The semicircular growth lines suggest that the sclerites may have grown unidirectionally towards the aperture of the calyx, although this interpretation may require further evidence to be conclusive.

## Discussion

**Figure 4:**
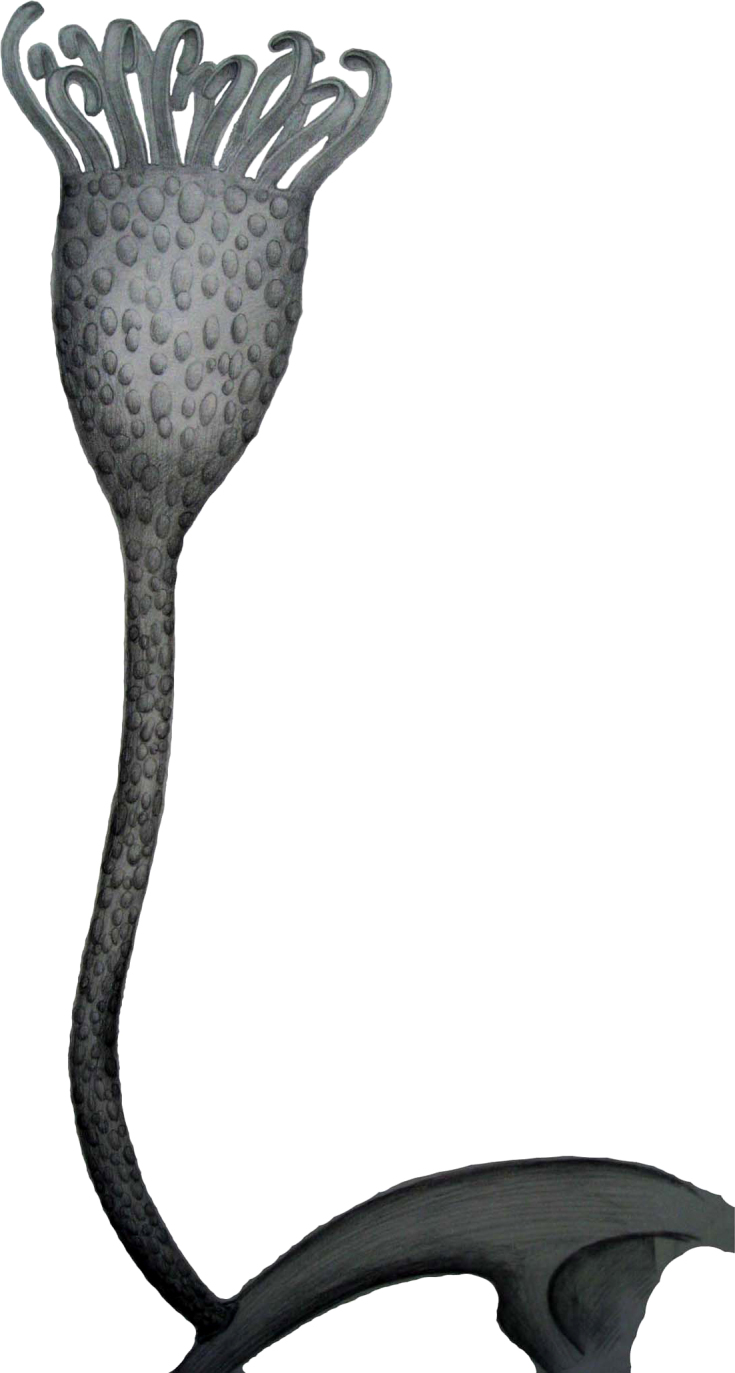
Reconstruction of *Cotyledion tylodes* Luo & Hu 1999 in life position.

The first description of the enigmatic *Cotyledion* was based on only two incomplete specimens, and it was accordingly referred to Problematica^6^ on account of the paucity of characters. Later, an additional well preserved specimen distinctly revealed a circle of tentacles around the upper margin of the calyx^12^, suggesting an uncertain tentaculate affinity of *Cotyledion.* Clausen et al.^11^ re-examined 27 specimens of *Cotyledion* and concluded that it belonged in the stem group of cnidarians. Our interpretations are based on observations of approximately 400 new specimens collected by the work-team of the Early Life Institute (Prefix: ELI). Well-preserved specimens provide considerable new morphological and anatomical data that form the basis for a major reconsideration of the structure, function and affinities of this peculiar tentaculate animal. The new material shows that *Cotyledion* was a solitary, sessile and benthic, tentaculate presumed suspension feeder (Fig. 4). Its body consisted of a lower stalk that normally attached (often gregariously) to hard bioclasts, and an upper cup-shaped calyx that contained a U-shaped gut and a tentacle crown that surrounded the mouth and anus (Figs. 1, 2). The presence of a U-shaped digestive tract unequivocally rule out an affinity of *Cotyledion* with the cnidarians^11^, but rather points to its inclusion in the sessile lophotrochozoans^13^,^14^. In contrast filter-feeding deuterostomes usually have bifurcating tentacles^15^.

The Lophotrochozoa^13^,^16^, represents a clade of remarkably diverse and anatomically disparate spiralian animals, comprising a number of animal phyla, specially Annelida, Mollusca and several sessile tentaculate groups (Bryozoa, Cycliophora, Brachiopoda, Entoprocta, Phoronida)^10^. Anatomically, the Entoprocta differs markedly from other sessile lophophorates in that both the mouth and anus lie inside the “crown” of tentacles. Not least because of differences in cleavage patterns and body cavities^17^, the Entoprocta is usually placed outside the Superphylum Lophophorata^18^. Recently, molecular phylogenies confirm that the entoprocts are monophyletic^19^ and invariably nest within the Lophotrochozoa, with recent results suggesting they are sister group to the cycliophorans and with weak support for forming the Polyzoa together with the bryozoans^14^,^16^,^20^.

Despite the relatively stable composition of the Lophotrochozoa, the precise internal relationships of the clade, and its morphological origins remains obscure, largely owing to the widely disparate nature of the phyla contained. As a result, reconstruction of the evolution of fossil stem-groups to extant crown groups is a necessary step to understanding this large group's early history. Such information has largely relied on exceptionally preserved fossils, such as from the Burgess Shale and Chengjiang^15^,^21^. Exceptionally preserved tentaculate fossils including eldoniids^22^, *Phlogites*^6^ and the recently reported *Herpetogaster*^15^, commonly characterized by a coiled intestine and paired bifurcated tentacles that encircle the mouth but not anus, are best referred to primitive deuterostomes rather than any lophotrochozoans^15^, let alone entoprocts typified by unbranched tentacles surrounding a mouth and anus in a whole^5^. Although originally compared to entoprocts^3^,^4^, the affinities of *Dinomischus* were later questioned owing to its lack of pliable and foldable tentacles^2^ and any further information on its interior anatomy^5^,^15^,^23^.

Although the exact phylogenetic placement of *Cotyledion* is still open to some speculation, the discovery of a U-shaped gut represents a significant progress in the understanding of these enigmatic Cambrian fossils, and demonstrates a bilaterian, lophophorate affinity of *Cotyledion* with certainty. The morphology (a U-shaped gut with a mouth and anus enclosed within a circle of tentacles) and sessile lifestyle of *Cotyledion* is compatible with an entoproct affinity^1^, and accords well with the recent suggestion based on molecular analyses that the entoproct ancestor had a “marine, probably solitary, epizoic adult stage”^19^. In addition, there is some evidence that the tentacles may have been contractible and could have been retracted into the membranous band where they originate from (Fig. S4a–c), suggesting some degree of retractability of the tentacles that corresponds functionally and structurally to that seen in those of the extant entoprocts^1^. Other entoproct characters include presence of a slit-like mouth and funnel-like buccal cavity (Fig. 1). By contrast, all extant solitary entoprocts are relatively diminutive, with height of individual zooids ranging from 0.3 to 30 mm and that of calyxes between 0.2 and 1.2 mm^1^, markedly less than the average calyx (4.3–42 mm) and individual height (8–56 mm) of *Cotyledion* (Fig. S1, Table S1). In addition, recent entoprocts are pseudocoelomate, with the cavity surrounding the calycal organs and extending into the stalk in-filled by a hydrostatic skeleton of loose mesenchyme cell or narrow primary body cavity^1^.

It is notable that a significant number of animals that arose in Cambrian explosion have combinations of very unfamiliar morphologies and anatomies^21^,^24^, not encountered in extant phyla. Nevertheless, the key utility of fossils in systematics is not supposedly insuperable difference, but shared similarities or homologous characters. Even when weak, such characters provide provisional support for particular stem-group placement of enigmatic taxa such as *Cotyledion* and assist in reconstruction the sequence of character acquisition in the Cambrian, and indeed can polarize hypothesis of character loss or gain in related derived extant crown groups. Thus, although *Cotyledion* i) is larger; ii) apparently possesses a more extensive body cavity; iii) has an external skeletal organization, it nevertheless shares features with entoprocts such as the holdfast + stalk + calyx bearing tentacles surrounding both mouth and anus, both linked by a U-shaped gut. Together, these are features possessed only by the extant entoprocts, and - despite the notable differences – suggest a placement of *Cotyledion* within the entoproct stem-group.

If correctly interpreted, this placement of *Cotyledion* has several novel implications for lophotrochozoans. Although living entoprocts are markedly smaller in adult size than *Cotyledion*, the size decrease could be taken as a result of miniaturization that is a widespread phenomenon in animals^25^. It is well known that miniaturization involves not only small body size, but also consequent and often dramatic effects of extreme size reduction on anatomy, ecology and life history^25^. Such consequences were well exemplified in *Cotyledion*. Along the stalk of *Cotyledion* (Fig. 1a, b; Fig. S4g), there is a central lineation of darkish color suggesting a central canal, interpreted here as an extension of the calycal cavity. Given that recent molecular results place the entoprocts within a clade of coelomates^14^,^16^, it is possible that the body cavity of *Cotyledion* was indeed a coelom. If so, the loss of a coelom may represent an apomorphic character in living entoprocts, since reduction and structural simplification is a common effect of miniaturization^25^, and an adaptation that may be associated with a change to colonial life. Moreover, the body wall of living entoprocts is very thin, even transparent on the calyx, solely covered by a glycoprotein cuticle with a trace of chitin^25^. By contrast, the outer body surface of *Cotyledion* hosts numerous pronounced sclerites that presumably stiffened the calyx or stalk (Fig. 3,4). Despite providing protection from durophagy^26^, the strongly armoured scleritome of *Cotyledion* might thus have precluded the organism from performing the flexible “nodding” motions as seen in recent entoprocts. The single-layered epithelial body wall in extant entoprocts likely represents another consequence of size decrease. It is apparent that *Cotyledion* had a solitary lifestyle and was attached to the exoskeletons of other organisms (Fig. 1a, c; Fig. S4c, e-g). Taking into account the evolutionary changes of the characters discussed above, solitary life could be a plesiomorphic state in contrast to the colonial one of modern entoprocts. The assumption is supported by the younger colonial entoprocts, so far known from the upper Jurassic rocks in Great Britain^2^. If so, the interpretation of *Cotyledion* among stem entoprocts demonstrates that a stolon as attachment structure is an apomorphy for the Order Coloniales within the Entoprocta^19^. The Cambrian (Stage 3) fossils discussed here are, therefore, a very significant extension of the geological range of total group entoprocts, set a minimum time frame for the divergence of entoprocts from other lophotrochozoans, and first shed light on their scleritomous body plan (Fig. 4).

The cuticular sclerites represent the most unique feature of *Cotyledion*. The discovery that *Cotyledion* is allied to entoprocts demonstrates that entoprocts developed distinctive armoured bodies early in their history. Comparable to *Cotyledion*, armoured scleritome-bearing bodyplans were also known from stem groups of annelids^27^, molluscs^21^,^28^,^29^, brachiopods^8^ and phoronids^9^, presumably thought as evidence of a close affinity between lophotrochozoans. Abundant varieties of sclerites commonly found in Cambrian SSF assemblages such as siphogonuchitids, sachitids and halkieriids have been associated with the stem groups of various branches of the lophotrochozoan tree (i.e. Mollusca and Annelida)^21^,^27^,^28^,^29^, and the phosphatic tommotiids have been placed in the stem groups of the Phoronida^9^ and Brachiopoda^7^,^8^. In terms of morphology, the scleritome of *Cotyledion* (particulary in the stalk) is most comparable to the tommotiid *Eccentrotheca*^9^,^30^, which has a tubular articulated scleritome composed of highly variable phosphatic sclerites. However, the original mineralogy of the sclerites in *Cotyledion* cannot be ascertained, and although there is some evidence for accretionary growth, the detailed shape and loose configuration of the scleritome of *Cotyledion* clearly differs from that of the tightly interlocking and organized configuration of *Eccentrotheca*. In *Eccentrotheca*, the sclerites are arranged in transverse, ring-shaped units that are eventually fused during ontogeny^9^,^30^. This contrasts sharply with the roughly longitudinal arrangement and universal separation of the sclerites in *Cotyledion*. The sclerites of *Cotyledion* (Fig. 3) also differ from those of *Eccentrotheca* and other tommotiids by the existence of a unidirectional mode of growth (Fig. 3f). Based on these differences, *Cotyledion* cannot be included in the tommotiids and the structural similarities of the stalk in *Cotyledion* with the scleritome of *Eccentrotheca* are superficial. The presence of external sclerites in the stem entoproct *Cotyledion* as well as in the proposed stem groups of phoronids^9^, brachiopods^7^,^8^, annelids^27^ and molluscs^21^,^28^,^29^ suggests that sclerites themselves are distributed more widely than thought before, and may even be plesiomorphic for the Lophotrochozoa. Despite the differences in organization, growth and composition, the discovery of *Cotyledion* as a sclerite-bearing entoproct strengthens the view that the stems of many of lophotrochozoan phyla may be traced within the early Cambrian Small Skeletal Fauna (SSF).

## Methods

All the material of *Cotyledion* was collected from the Yu'anshan Member (*Eoredlichia*-*Wutingaspis* Zone) of the upper part of the Lower Cambrian Heilinpu Formation by the team from the Early Life Institute (ELI) of Northwest University, Xi'an, China. The fossils were examined by Z. Zhang with a binocular Olympus Zoom Steromicroscope and photographed with a Nikon camera mounted on a photomicrographic system, with different illuminations for particular views when high contrast images are required. Measurements were directly made by means of a millimeter ruler. The SEM microphotographs of uncoated fossils were taken at Stage Key Laboratory of Continental Dynamics, Northwest University, Xi'an and some assisted by Leonid Popov at National Museum of Wales, Cardiff, UK.

### Nomenclatural acts

This published work and the nomenclatural acts it contains have been registered in ZooBank, the proposed online registration system for the International Code of Zoological Nomenclature (ICZN). The ZooBank LSIDs (Life Science Identifiers) can be resolved and the associated information viewed through any standard web browser by appending the LSID to the prefix ‘http://zoobank.org/'. The LSID for this publication is: urn:lsid:zoobank.org:pub:4810D25C-02BB-4674-B508-5D5D67DEF9EE, and urn:lsid:zoobank.org:act:51669C19-D7EC-469D-A537-DDA51655E4FA.

## Author Contributions

All authors participated in the discussion and analysis of these fossils. Collection of material was made by Chinese members (ELI). Z.F. Zhang designed the study and made statistics of these fossils. Z.F. Zhang, L.E. Holmer, G.A. Brock and C.B. Skovsted prepared the earlier manuscript, and G. Budd corrected the English and improved the final version. Z.F. Zhang, D.J. Fu and J. Han made the artistic reconstruction of *Cotyledion*.

## Supplementary Material

Supplementary Information Supplementary information
