# Supplementary material for: A sclerite-bearing stem group entoproct from the early Cambrian and its implications
Source: Sci Rep. 2013 Jan 17;3:1066. doi: 10.1038/srep01066 (PMC3548229; doi:10.1038/srep01066)

SUPPLEMENTARY INFORMATION

A sclerite-bearing stem group entoproct from the early Cambrian and its implications

Zhifei Zhang[1,5*], Lars E. Holmer[2], Christian B. Skovsted[3], Glenn A. Brock[4], Graham E. Budd[2], Dongjing Fu[1], Xingliang Zhang[1], Degan Shu[1], Jian Han[1], Jianni Liu[1], Haizhou Wang[2], Aodhan Butler[2] & Guoxiang LI [5]

[1]Early Life Institute, State Key Laboratory of Continental Dynamics, Northwest University, Xi'an, 710069, China

[2]Uppsala University, Department of Earth Sciences, Palaeobiology, Villavägen 16, SE-752 36, Uppsala, Sweden

[3]Department of Palaeozoology, Swedish Museum of Natural History, Box 50007, SE-104 05, Stockholm, Sweden

[4]Department of Biological Sciences, Macquarie University, New South Wales 2109, Australia

[5]LPS, Nanjing Institute of Geology and Palaeontology, Chinese Academy of Sciences, Nanjing, 210008, China.

[*] Correspondence should be addressed to elizf@nwu.edu.cn or zhangelle@126.com

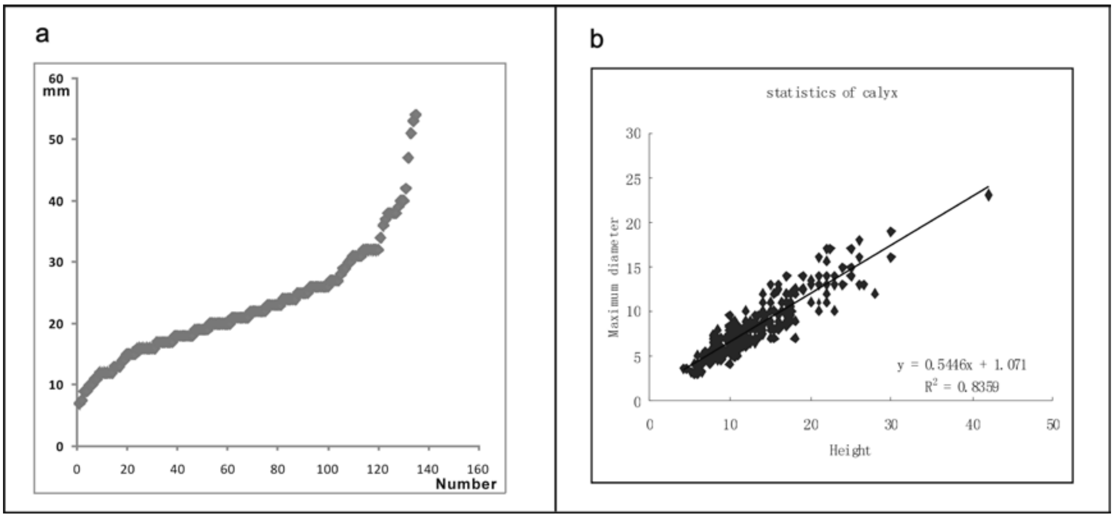

Fig. S1. Size statistics for the stem entoproct *Cotyledion tylodes* Luo et Hu 1999 from the Chengjiang fauna. **a,** body height variations of *Cotyledion*; **b,** plots of maximum height verus diameter of calyx in *Cotyledion* based on ELI collection from the early Cambrian Chengjiang fauna of China.

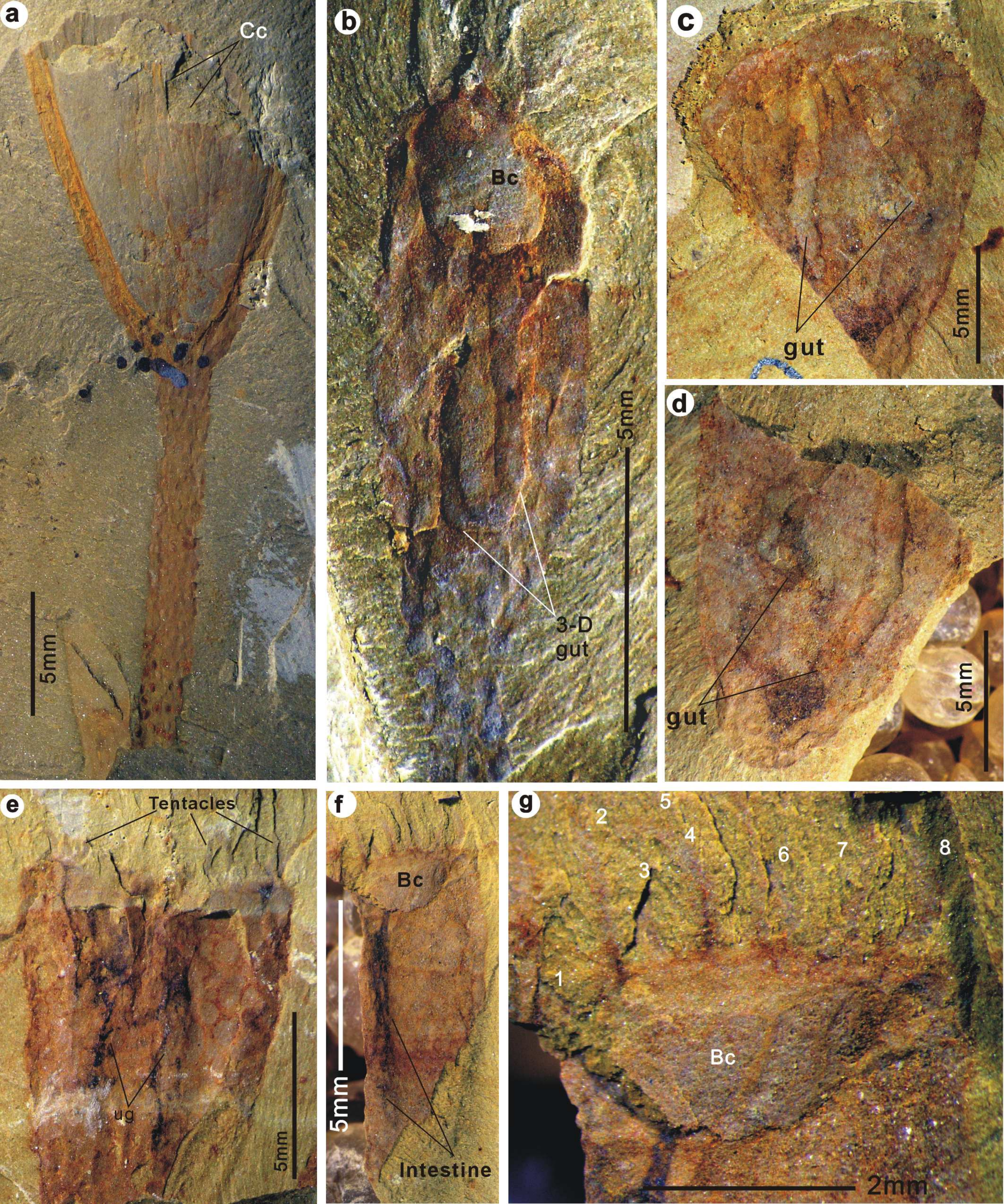
a
Cc
5mm
b
Bc
5mm
3-D
gut
c
gut
5mm
d
gut
5mm
e
Tentacles
ug
5mm
f
Bc
5mm
Intestine
g
1
2
3
4
5
6
7
8
Bc
2mm

Fig. S2. The early Cambrian Chengjiang stem entoproct *Cotyledion tylodes* Luo et Hu 1999. **a**, ELI-C-0367B, Internal mould of mud-filled calyx cavity (Cc); **b**, ELI-C-0045A, an anterolaterally crushed specimen showing mud-infilled buccal cavity (Bc) and 3-D preserved gut; **c**, ELI-C-0346A, showing the mud-filled U-shaped digestive tract with pronounced relief; **d**, Counterpart of c; **e**, ELI-C-0312B, internal view of a calyx, showing the U-shaped impression of gut (ug) and basal impressions of sclerites on the internal wall of calyx; note the membrane band with tentacles; **f**, ELI-C-0296B, general view of a specimen squeezed in a mouth-anus direction; **g**, Details of the tentacles in f, note the mud-filled slit-like mouth (Bc).

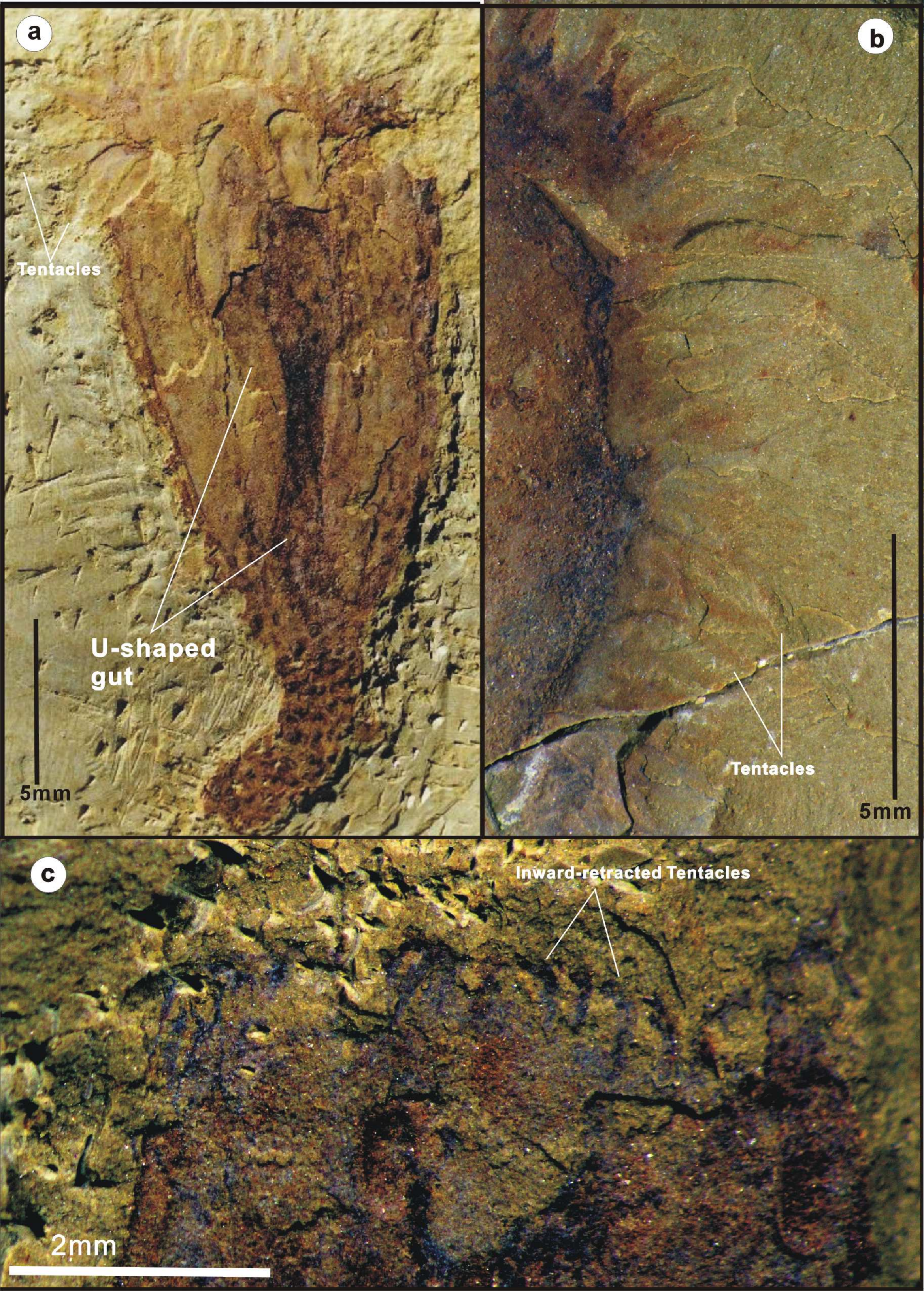
a
Tentacles
U-shaped
gut
5mm
b
Tentacles
5mm
c
Inward-retracted Tentacles
2mm

Fig. S3. Showing the tentacles of *Cotyledion* from the Chengjiang fauna. **a**, a anterior-laterally squeezed specimens with a circle of extended tentacles superbly preserved[11]; **b**, ELI-C 0375B, enlargement of the tentacles. **C**, ELI-C 0025A, close-up view of the inward-retracted and contracted tentacles.

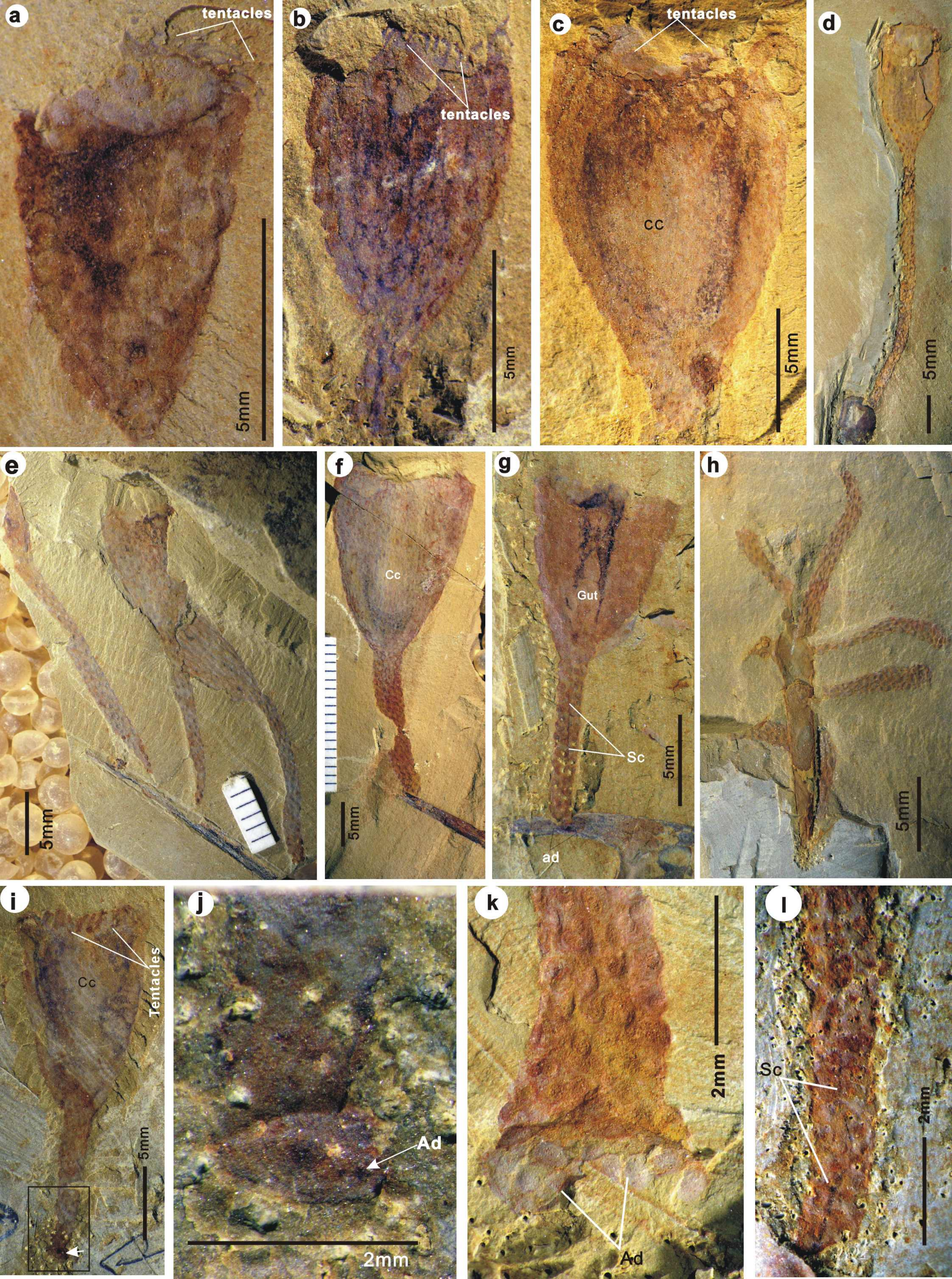

a
tentacles
5mm
b
tentacles
5mm
c
tentacles
cc
5mm
d
5mm
e
5mm
f
Cc
5mm
g
Gut
Sc
5mm
ad
h
5mm
i
Tentacles
Cc
5mm
j
Ad
2mm
k
2mm
Ad
l
Sc
2mm

Fig. S4. Additional specimens of *Cotyledion tylodes* Luo & Hu 1999 from the Haikou and Erjie areas of Yunnan, China. **a**, ELI-C-0326, top view of the calyx with tentacles; **b**, **c**, ELI-C-0025A and 0034A, laterally compressed two specimens with contracted tentacle crown encircling retracted centrally pointing tentacles; **d**, ELI-C-0370, a specimen with long stalk attached to a trilobite, putatively crushed in mouth-anus direction; **e**, ELI-C-0346A, 3 individuals of *Cotyledion* commonly attached to a cheek of trilobite; **f**, ELI-C-0337A, a flattened specimen, showing the calyx cavity (Cc) and stalk attached to a trilobite spine; **g**, ELI-C-0194B, note the coelomic cavity of stalk (Sc); **h**, ELI-C-0346A, a gregarious population of *Cotyledion tylodes* attached by stalks in common to a single hyolith shell; **i**, ELI-C-0250A, a flattened impression of *Cotyledion*, noting the tentacles on the top calyx and knob-like holdfast at the end of the stalk. **j**, ELI-C-0250A, enlarged view of the knob-shaped attachment disc (Ad) in i. **k**, ELI-C-0333B, showing the distal attachment disc (Ad) of stalk; **l,** ELI-C-0352A, close-up view of the coelomic canal in the stalk.

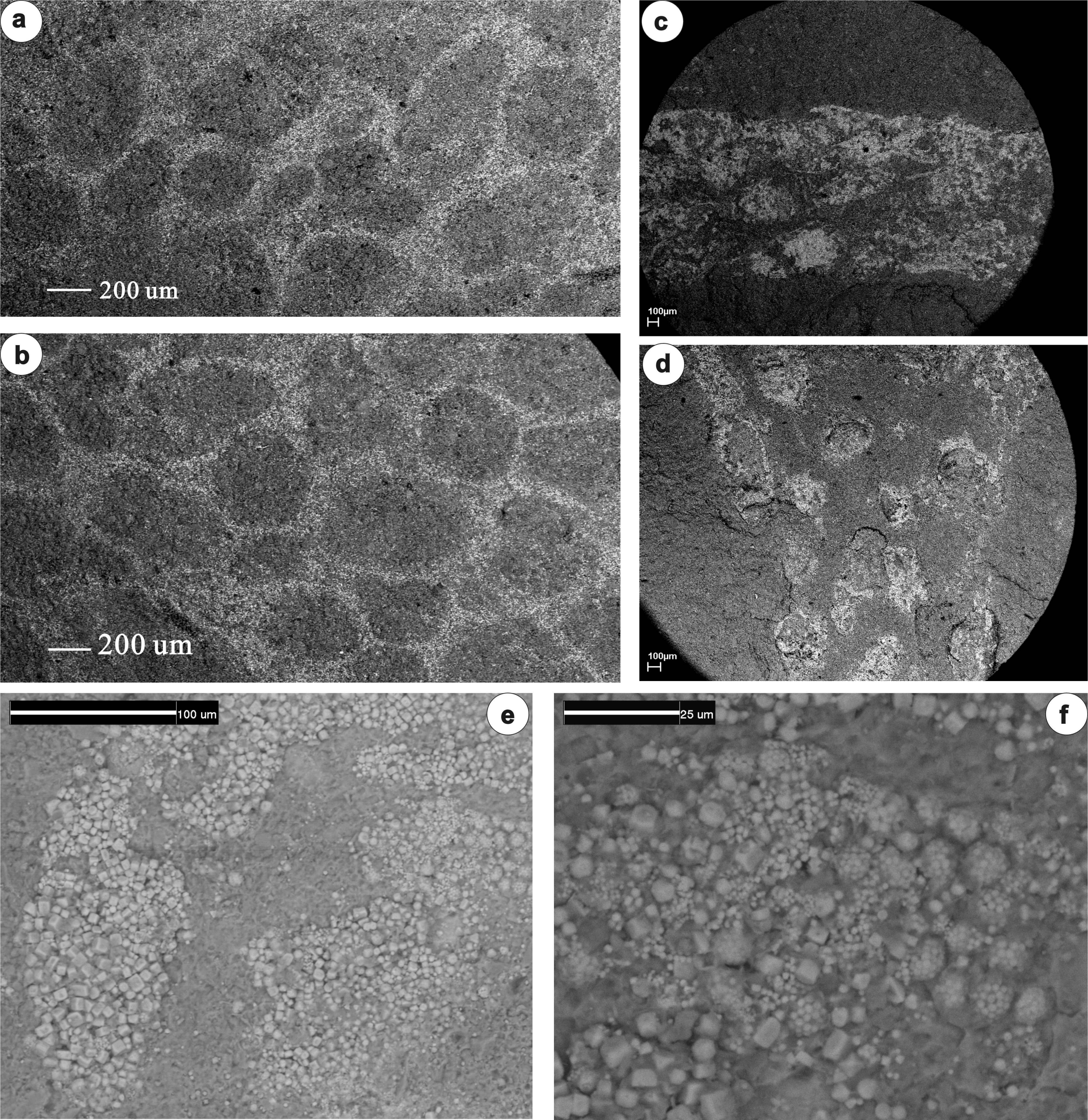
a
200 um
b
200 um
c
100µm
d
100µm
100 um
e
25 um
f

Fig. S5. BSEM examination of the sclerites of *Cotyledion* Luo et Hu 1999 from Chengjiang. **a**, ELI-C-0359A, interior mould of calyx, showing the pyrite preservation of sclerite impressions; **b**, BSEM of ELI-C-0359A; interior mould of calyx， showing calyx sclerite impressions netted together; **c,** ELI-C-0369, BSEM picture of stem sclerite impressions of *Cotyledion*; **d**, BSEM picture of transition section between the Calyx and stem of ELI-C-0370. **e** and **f**, Enlarged and detailed view of pseudomorphed framboidal pyrite on the sclerite of d.

Table S1. Statistics for *Cotyledion* based on ELI collection from the Chengjiang fauna, Yunnan, South China. All measurements are in mm. The following abbreviations are used: Lc, the height of calyx; Ls, the length of stalk; Wc, the diameter of calyx; Ws, the diameter of stalk. H, the body height of *Cotyledion* based on ELI collection.

	Lc	Ls	Wc	Ws	Lc/Wc	Lc/Ls	Ws/Wc	H
Count	292	120	300	203	293	70	194	107
Min	4. 3	2. 7	3	0. 8	1. 05	0. 33	0. 11	8
Max	42	38	23	3	2. 57	2. 4	0. 5	56
Mean	13. 3	15. 44	8. 32	1. 65	1. 61	0. 78	0. 23	26. 4
SD	5. 55	6. 47	3. 2	0. 39	0. 26	0. 33	0. 06	0. 23

Table S2. EDX analysis of the sclerites of *Cotyledion*, showing the high concentration of iron oxide with fewer amounts of phosphorus; note the moderate amount of aluminosilictes from the surrounding matrix.

Elements	Weight(%)	Atom(%)
Mg K	1.15	1.25
Al K	7.15	6.96
Si K	9.19	8.59
P K	0.88	0.74
K K	1.16	0.78
Fe K	43.01	20.22
O	37.45	61.46
Total	100.00	

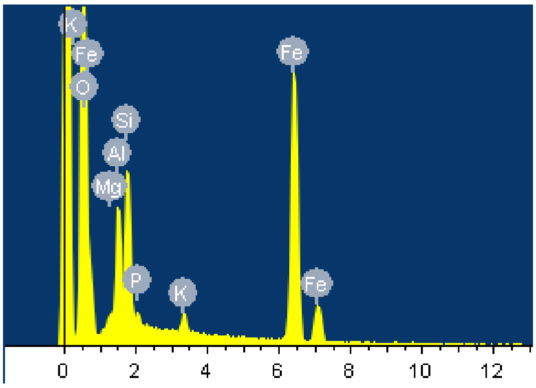

Supplement: Supplementary Information [file srep01066-s1.pdf]
